# Tyramine and Amyloid Beta 42: A Toxic Synergy

**DOI:** 10.3390/biomedicines8060145

**Published:** 2020-05-30

**Authors:** Sudip Dhakal, Ian Macreadie

**Affiliations:** School of Science, RMIT University, Bundoora, VIC 3083, Australia; sudip.dhakal@rmit.edu.au

**Keywords:** tyramine, amyloid-beta, Alzheimer’s disease, oxidative stress, yeast, petite mutant

## Abstract

Implicated in various diseases including Parkinson’s disease, Huntington’s disease, migraines, schizophrenia and increased blood pressure, tyramine plays a crucial role as a neurotransmitter in the synaptic cleft by reducing serotonergic and dopaminergic signaling through a trace amine-associated receptor (TAAR1). There appear to be no studies investigating a connection of tyramine to Alzheimer’s disease. This study aimed to examine whether tyramine could be involved in AD pathology by using *Saccharomyces cerevisiae* expressing Aβ42. *S. cerevisiae* cells producing native Aβ42 were treated with different concentrations of tyramine, and the production of reactive oxygen species (ROS) was evaluated using flow cytometric cell analysis. There was dose-dependent ROS generation in wild-type yeast cells with tyramine. In yeast producing Aβ42, ROS levels generated were significantly higher than in controls, suggesting a synergistic toxicity of Aβ42 and tyramine. The addition of exogenous reduced glutathione (GSH) was found to rescue the cells with increased ROS, indicating depletion of intracellular GSH due to tyramine and Aβ42. Additionally, tyramine inhibited the respiratory growth of yeast cells producing GFP-Aβ42, while there was no growth inhibition when cells were producing GFP. Tyramine was also demonstrated to cause increased mitochondrial DNA damage, resulting in the formation of petite mutants that lack respiratory function. These findings indicate that there can be a detrimental synergy between Aβ42 and tyramine, which could be considered in Alzheimer’s disease. This work also demonstrates the utility of yeast as a model for studying toxic agents such as Aβ42, tyramine, and agents that might exacerbate AD pathology.

## 1. Introduction

**Figure 1:**
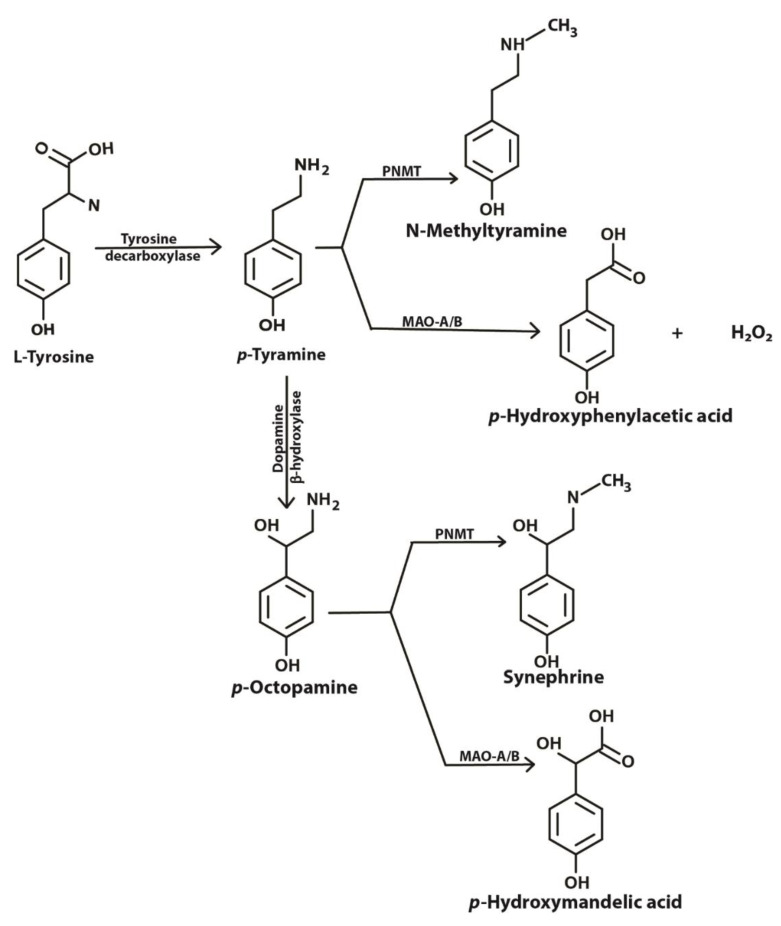
Metabolic pathway showing biosynthesis and fate of tyramine inside a cell. PNMT, phenylethanolamine-N-methyl transferase; MAO-A/B, monoamine oxidase A or B (adapted from [1] under license agreement CC BY-NC 4.0).

Tyramine is an important amine obtained in the diet and also produced in the brain and other tissues from its precursor tyrosine (Figure 1). Binding of tyramine to trace amine-associated receptor 1 (TAAR1), a G-protein coupled receptor, in the synaptic cleft reduces the activity of serotonergic and dopaminergic receptors [1]. TAAR1 has been reported to be involved in emotions, reward and cognition, and modulates monoaminergic and glutamatergic neurotransmissions through signaling activation of adenylyl cyclase activity [2]. TAAR1 has been found to heterodimerize with the dopamine receptor 2, which is thought to silence glycogen synthase kinase 3 beta (GSK3β) implicated in various mental disorders [3]. Imbalances in levels of tyramine have been reported to be associated with Parkinson’s disease, depression, schizophrenia, migraines and increased blood pressure [1,4,5,6]. In mammalian cells, tyramine has been found to be toxic [7], to cause oxidative damage [4] and to have effects on mitochondrial respiratory function [8].

Tyramine can be converted to octopamine and *p*-hydroxyphenyl acetate by the action of dopamine beta-hydroxylase and monoamine oxidase (MAO), respectively (Figure 1). Located in the membrane of the mitochondrion, MAO (specifically MAO-A) deaminates tyramine, producing hydrogen peroxide as a byproduct [9]. The presence of reduced glutathione (GSH) inside the mitochondrion helps overcome the peroxide toxicity. However, increased MAO activity in the mitochondria may result in the depletion of cellular reduced GSH. Intramitochondrial GSH levels are crucial for the maintenance of the mitochondrial DNA (mtDNA) as it is not protected with histones [8]. mtDNA encodes some genes that are required for the survival of the cell. This critical role of MAO and redox balance in mitochondria is paramount to protecting a cell. 

Blood-to-brain transport of tyramine is reported to be in low amounts; however, the clearance from brain to blood is higher. A study in rats demonstrated the involvement of brain parenchyma at the blood cerebrospinal fluid barrier in clearance of tyramine from brain to blood [10]. Despite extensive research in this biogenic amine’s functions, limited studies have been done to address tyramine’s involvement in Alzheimer’s Disease (AD). This could probably be due to the lack of appropriate models for such study. This report investigates the effect of tyramine in the presence of amyloid-beta in a yeast cell model.

It is widely understood that the amyloid-beta peptide of 42 amino acids (Aβ42) is associated with AD [11]. Aβ42 has been found to increase oxidative damage in cells and has been thought of as a major cause of the disease [11,12]. Amyloid plaques in human brains are the hallmark for the diagnosis of the disease [13]. However, other factors can also be involved in the disease progression as shown by the extensive epidemiology study on simvastatin, where it lowers the incidence of AD by half [14]. Simvastatin has been recently reported to reduce Aβ42 from yeast cells, implying that its action in people is through reduction of Aβ42 [15]. Diets are often implicated in AD, and various studies have shown protective effects of bioactives to neuronal cells. However, unlike prescription drug studies, it is hard to obtain rigorous data to support dietary interventions [16]. 

We have developed yeast-based models for studying the effects of compounds that aid the clearance or block the toxicity associated with Aβ42 [15,17,18]. In this report, the yeast model is also proven to be a convenient model to study compounds that exacerbate Aβ42 toxicity. This report investigated the effect of tyramine in normal yeast cells and genetically modified yeast cells expressing GFP-tagged Aβ42 and native Aβ42. Here, we show a synergistic effect of tyramine on Aβ42-mediated respiratory growth inhibition indicating enhanced irreversible damage to the mtDNA. 

## 2. Experimental Section

### 2.1. Yeast Strains, Plasmids and Growth Media 

The *Saccharomyces cerevisiae* yeast strain BY4743 *(MATa*/*α his3∆1*/*his3∆1*, *LYS2*/*lys2∆0 met15∆0*/*MET15 ura3∆0*/*ura3∆0 leu2∆0*/*leu2∆0*) was the host strain used in this study. The plasmids p416GPD.GFP.Aβ, p416GPD.GFP, pYEX.Aβ and pYEX.BX were transformed into the host strain, BY4743 [15]. Strains are named BY4743 (p416GPD.GFP), BY4743 (p416GPD.GFP.Aβ), BY4743 (pYEX.Aβ) and BY4743 (pYEX.BX) after transformation with corresponding plasmid vectors.

Minimal selective media used for the selection and growth of the transformants were composed of yeast nitrogen base without amino acids (0.67%), dextrose (2%): leucine (20 mg/L), histidine (20 mg/L) and uracil (20 mg/L) were supplemented in the media for auxotrophic requirements of the transformants where required. Yeast extract peptone dextrose (YEPD) and Yeast extract peptone ethanol (YEPE) media supplemented with different concentrations of tyramine were used for the respiratory growth inhibition assays (Section 2.3). YEPD medium was composed of yeast extract (1%), dextrose (2%) and peptone (2%). YEPE was prepared like YEPD, but ethanol was the carbon source in the place of dextrose. Solidified media were prepared by the addition of 2% agar. YEPDE was YEPE with 0.1% dextrose added.

### 2.2. Tyramine-Induced ROS Detection in Yeast

Freshly prepared 2′,7′-dichlorodihydrofluorescein diacetate (H_2_DCF-DA) was used for the estimation of ROS in cells treated with different concentrations of tyramine [19]. The presence of ROS was determined by the conversion of H_2_DCF-DA to DCF, which fluoresces at 530 nm when excited at 488 nm. Overnight culture of BY4743 cells were harvested and freshly grown for 2–3 h at 30 °C for the cells to achieve exponential growth phase. The fresh cells were treated with different concentrations of tyramine. During the treatment, H_2_DCF-DA was also added at a final concentration of 10 µg/mL. To determine if reduced glutathione can rescue the ROS generated by tyramine, 5 mM glutathione was added with the 5 mM tyramine in a separate tube with H_2_DCF-DA. Cells were incubated in the dark at 30 °C for 2 h. After incubation, yeast cells were harvested and washed with sterile water twice. Cells were incubated for subsequent growth in media for 1 h. At the completion of the final incubation, cells were washed with PBS and analyzed for green fluorescence using a flow cytometer (10,000 events per sample). The green fluorescence emitted by blue laser at 488 nm was measured using a 530/30 bandpass filter in a FACS Canto II flow cytometer (BD Life Sciences, San Jose, CA, USA). Controls included unstained cells, untreated cells and a positive control (H_2_O_2_ treatment) to configure the gating and analysis. Data obtained from flow cytometry were analyzed using Flow Jo Version 10.6.0 (BD Life Sciences, San Jose, CA, USA).

### 2.3. Respiratory Growth Inhibition Assay

Freshly grown *S. cerevisiae* BY4743 (p416GPD.GFP) and BY4743 (p416GPD.GFP.Aβ) transformants were examined for respiratory growth inhibition, with concentrations of tyramine ranging from 0 to 3 mM. Cell counts were obtained using an Automated Cell Scepter Counter (Merck Millipore, Bayswater, VIC, Australia). Equivalent transformants of both types were serially diluted (10-fold) in a 96-well plate using sterile water as diluent. Diluted cells were inoculated in fresh YEPD and YEPE plates supplemented with different concentrations of tyramine using a multi-pronged inoculator. Growth inhibition in both (YEPD and YEPE) plates were recorded and analyzed for significant differences in the two strains after incubating cells at 30 °C for 3–5 days.

### 2.4. Petite Frequency Determination

*S. cerevisiae* BY4743 (p416GPD.GFP) and BY4743 (p416GPD.GFP.Aβ) transformants were grown in YEPD with various concentrations of tyramine before being plated onto YEPDE plates at 30 °C for 3–5 days. The total colony forming units (CFU), larger colonies (grandes) and small colonies (petites) were counted to determine petite frequencies (as percentages).

### 2.5. Data Analysis

Data obtained in triplicate were analyzed using GraphPad Prism Version 7 (GraphPad Software, San Diego, CA, USA). Error bars represent the Standard Error of the Mean (SEM). One-way ANOVA and two-way ANOVA tests were used to analyze the data.

## 3. Results

### 3.1. Tyramine Enhances Reactive Oxygen Species (ROS) in Yeast Cells

**Figure 2:**
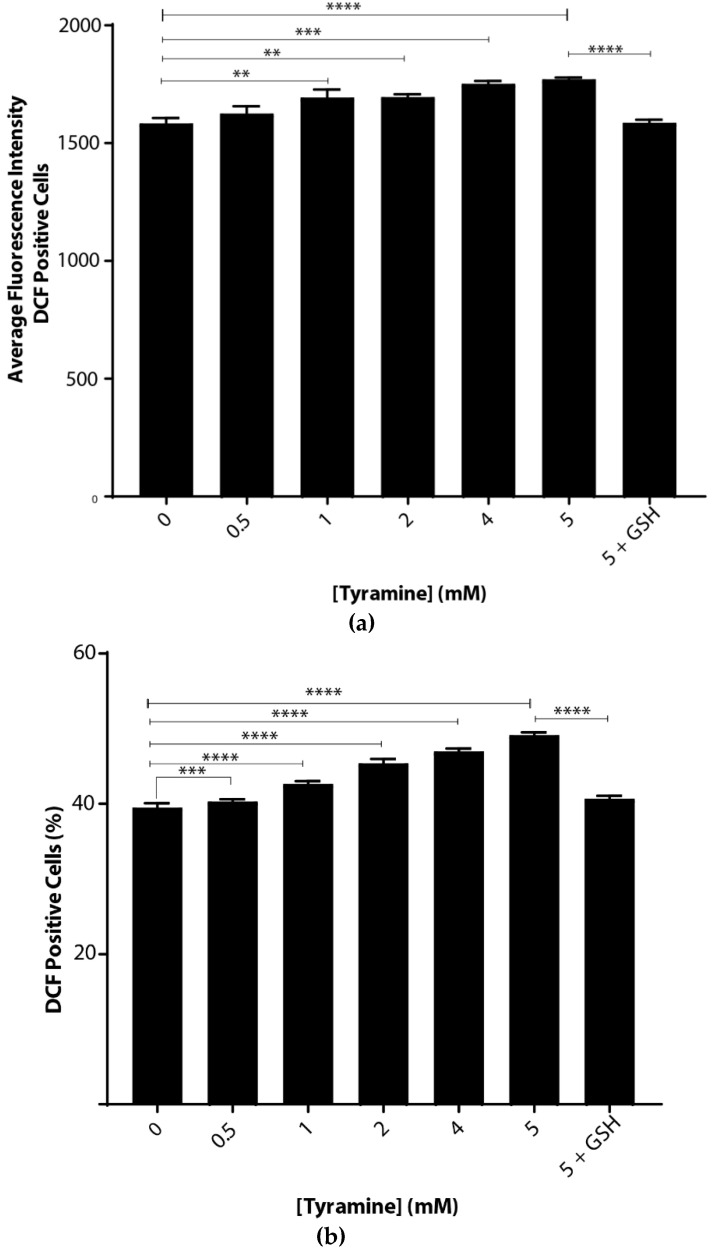
Tyramine dose-dependent ROS determination in *Saccharomyces* *cerevisiae* BY4743 using 2′,7′-dichlorodihydrofluorescein diacetate (H_2_DCFDA) staining. (**a**) Average single-cell fluorescence intensity of the dichlorofluorescein (DCF)-positive yeast cells at different concentrations of tyramine and (**b**) dichlorofluorescein (DCF)-positive cell counts after tyramine treatment. The concentration of 5 mM glutathione was used for rescue of the cells from oxidative stress. Values are from triplicates; values significantly different from 0 mM tyramine in one-way ANOVA, and values significantly different from 5 mM tyramine are indicated (** *p* < 0.01, *** *p* < 0.001 and **** *p* < 0.0001).

Oxidative stress caused by tyramine was examined by incubating *S. cerevisiae* BY4743 cells suspended in chemically defined yeast-minimal media. There were significant dose-dependent increases in ROS with tyramine treatment (Figure 2), implying that tyramine induced oxidative stress in cells. This increase was measured in two ways. First, as shown in Figure 2a, the average fluorescence intensity increased from the arbitrary value of 1528 to 1738. Second, the number of fluorescent cells increased from 39.8% to 51.3% (Figure 2b). At the highest tyramine level tested, 5 mM, the ROS induced by tyramine was fully reduced by the addition of reduced glutathione, as shown in Figure 2a,b. Thus, we conclude that tyramine is a strong inducer of reactive oxygen species.

### 3.2. Tyramine Exacerbates Oxidative Stress in Yeast Producing Native Aβ42

**Figure 3:**
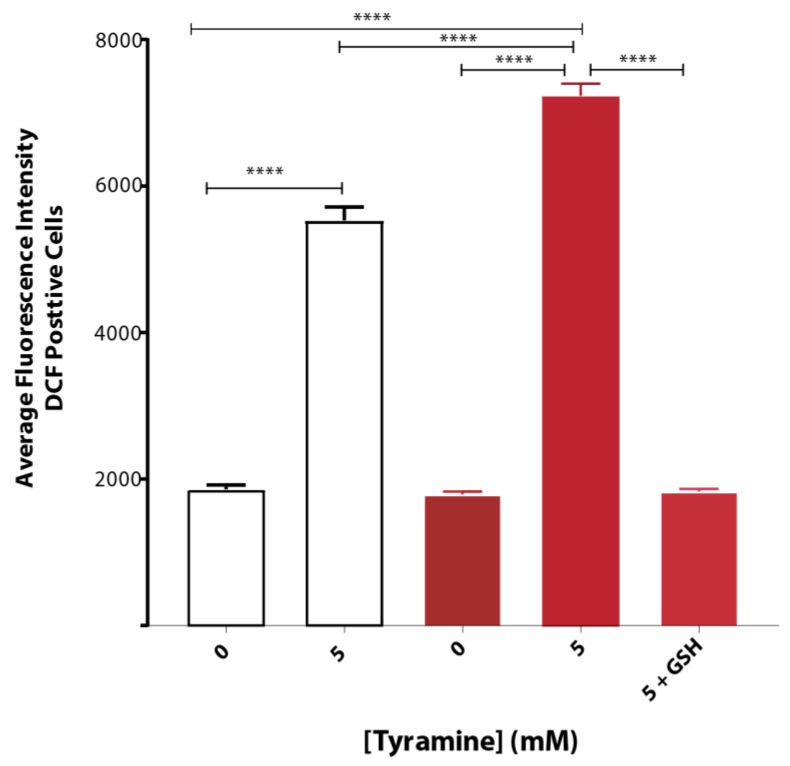
Oxidative stress of *S. cerevisiae* BY4743 (pYEX.BX) (white bar) and BY4743 (pYEX.Aβ) (red bar) transformants using H_2_DCFDA staining in presence of tyramine and rescue with 5 mM reduced glutathione. Values are from triplicates, and values significantly different in one-way ANOVA are indicated by asterisks ( **** *p* < 0.0001).

Tyramine induced ROS formation to a greater extent in yeast strain BY4743 (pYEX.Aβ) as compared to that of empty vector control BY4743 (pYEX.BX) demonstrated by H_2_DCFDA staining. The average fluorescence intensity of DCF-stained cells treated with 5 mM tyramine was overcome with 5 mM reduced glutathione (Figure 3). 

### 3.3. Tyramine Strongly Inhibits Respiratory Growth in the Presence of Aβ42

**Figure 4:**
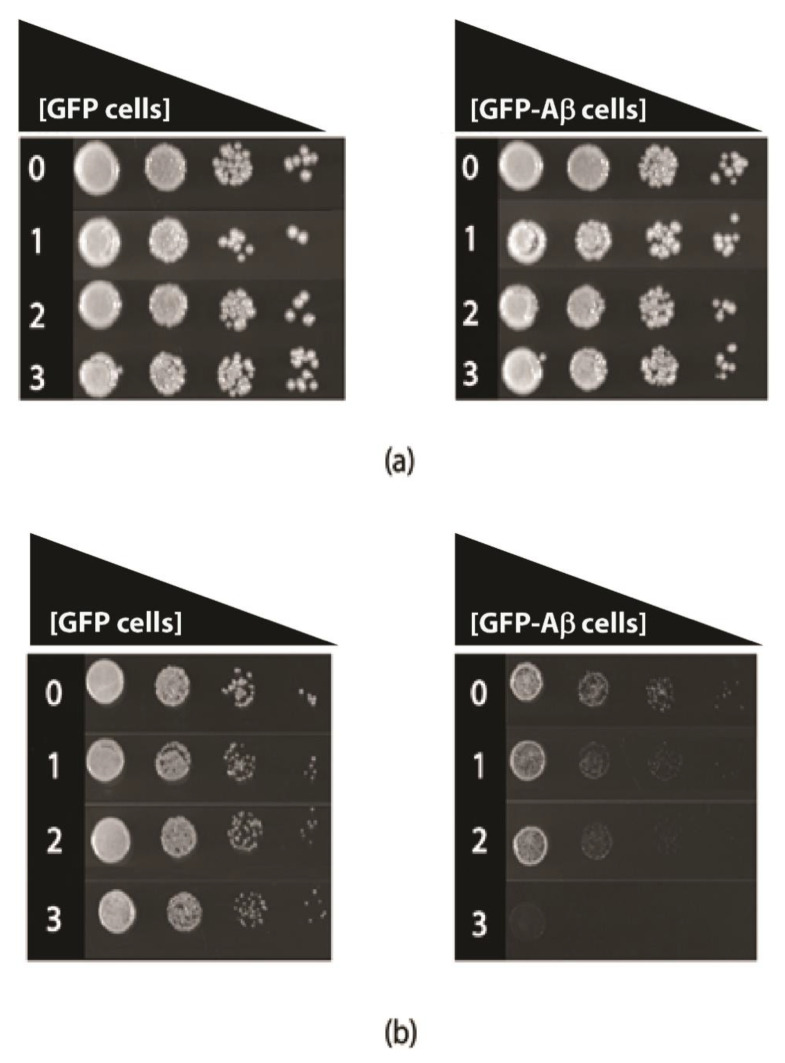
Growth of *S. cerevisiae* BY4743 (p416GPD.GFP) and BY4743 (p416GPD.GFP.Aβ) transformants on (**a**) Yeast extract peptone dextrose and (**b**) Yeast extract peptone ethanol media in the presence of 0, 1, 2 and 3 mM tyramine incubated at 30 °C for 3–5 days.

Tyramine toxicity was also examined in fermentative and respiratory growth assays, in transformed yeast cells that constitutively produce GFP-Aβ42 or GFP alone. The comparison of these constructs was made because the growth of the transformed cells is quite similar and robust [17]. In fermentative growth, with glucose as a carbon source (YEPD media), there was no major growth inhibition (Figure 4); however, respiratory growth with ethanol as the carbon source (YEPE media) was severely reduced in the presence of levels of tyramine, even as low as 1 mM (Figure 2) for GFP-Aβ42 transformants. At 3 mM concentration of tyramine, no growth of GFP-Aβ42 transformants was observed in YEPE media. However, GFP-producing yeast showed no growth inhibition with these concentrations of tyramine. Notably, tyramine has a major impact on mitochondrial respiratory function in cells expressing GFP-Aβ42, indicating a synergistic detrimental effect on respiration with a tyramine-plus-GFP-Aβ42 combination.

### 3.4. Tyramine Induces Petite Formation in GFP-Aβ42 Producing Yeast Cells

**Figure 5:**
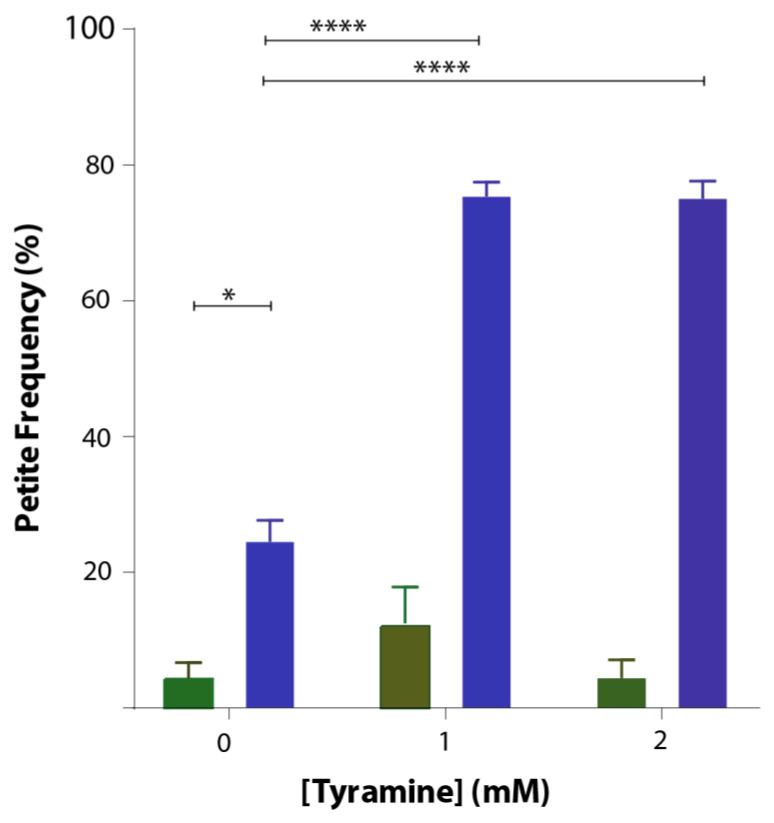
Petite frequency of *S. cerevisiae* BY4743 (p416GPD.GFP) (green bar) and BY4743 (p416GPD.GFP.Aβ) (blue bar) transformants treated with 0, 1 and 2 mM tyramine. Values are from triplicates, and values significantly different in two-way ANOVA are indicated by asterisks (* *p* < 0.05 and **** *p* < 0.0001).

*Saccharomyces cerevisiae* is an ideal organism to study not only effects on respiration but also effects on the mitochondrial genome. Deletions of the mitochondrial genome in portions or in entirety are not lethal in *S. cerevisiae* but lead to respiration-deficient mutant cells known as petites. Mitochondrial DNA damage can readily be studied in yeast. To study whether tyramine causes mitochondrial DNA damage, inducing petite formation, cells producing GFP and GFP-Aβ42 were grown in YEPD media with tyramine for 48 h. Cells were then spread out into solidified YEPDE media to determine the petite frequency. The petite frequency of GFP- and GFP-Aβ42-producing cells are shown in Figure 5. The data show significantly higher proportions of petites in transformants producing GFP-Aβ42. Results obtained also suggest that the concentration of 1 mM tyramine was enough for a statistically significant increase in the proportion of petites in transformants producing GFP-Aβ42.

## 4. Discussion

Tyramine was tested for its ability to enhance ROS signaling and its effect on respiratory growth of wild-type yeast cells and yeast producing different forms of Aβ42. Results obtained suggest that tyramine induces statistically significant levels of ROS in wild-type yeast in a dose-dependent manner. Interestingly, ROS generated in native Aβ42 transformant cells were statistically significant, showing a synergistic effect in the presence of tyramine. However, in both wild-type and transformant cells, reduced glutathione rescued the ROS indicated by reduced fluorescence. In addition, tyramine inhibited growth of GFP-Aβ42 cells in YEPE media in a dose-dependent manner, while no effect was observed for GFP transformants. This suggests synergistic toxicity of tyramine and Aβ42 in yeast mitochondria. Furthermore, the petite frequency in GFP-Aβ42 transformants was statistically significantly higher than in GFP cells. The presence of tyramine significantly increased the petite frequency of GFP-Aβ42, while there was no effect on GFP cells, suggesting a synergistic effect of tyramine with Aβ42. 

In this study, yeast cells provided an excellent model to study the toxic effects of Aβ42 in conjunction with tyramine. However, most of the previous studies involving yeast with Aβ42 focused more on finding compounds that can reduce Aβ42 toxicity [18]. Here, genetically modified yeast cells provide convenient models to embark on ideas involving mitochondrial dysfunction and regeneration as yeast can survive without mitochondrial function. 

Tyramine generally acts through the TAAR1 receptors in mammalian cells [20]. However, in yeast cells, TAAR1 or its orthologs are absent, which is why tyramine treatment in these cells can predict the intracellular effect of tyramine accumulation regardless of TAAR1 signaling. Inside mammalian cells, tyramine is a substrate for monoamine oxidases (types A and B). Yeast orthologs of MAO-A and MAO-B, referred to as polyamine oxidase, may act on the tyramine-producing hydrogen peroxide, increasing mitochondrial ROS levels. ROS induced by tyramine in healthy cells do not cause great damage to these yeast cells, as evident by the lower petite frequency. In contrast, yeast expressing Aβ42 underwent dose-dependent damage of the mtDNA, as demonstrated by nearly 80% petite generation with 1 mM and 3 mM tyramine treatments.

Studies suggest that there are only two mechanisms of how free radicals are generated inside mitochondria: first, the mitochondrial electron transport chain, and second, the MAO-A/B activity [8]. Due to its hydrophobic and aggregative nature, Aβ42 is thought to be membrane-bound where it is hypothesized to alter the membrane-associated electron transport chain, causing generation of free radicals [11,12], while tyramine is a substrate for MAO-A/B, causing ROS formation inside mitochondria [21]. Enhanced accumulation of free radicals inside mitochondria due to synergism of Aβ42 and tyramine might have depleted the available intramitochondrial GSH levels. In the oxidative microenvironment of mitochondria, transition metals like copper work together with free radicals in generating the single-stranded breaks and causing alterations in DNA sequences in the unprotected mitochondrial DNA, leading to irreversible permanent damage to mtDNA and generating petites [4]. Increased hydrogen peroxide may also be available for iron- and copper-mediated Fenton reactions to produce highly reactive hydroxyl radicals, enhancing the oxidative damage to the mitochondria [22,23]. Meanwhile, alteration of the tightly regulated brain distribution of these biometals can independently cause ROS formation [24]. Recovery of cells from high levels of ROS accumulation with externally added reduced glutathione strongly supports this finding. In humans, the combination of tyramine and Aβ42 could cause mitochondrial dysfunction and be lethal to neuronal cells.

Additionally, hyperactivated MAO-A has been reported to impair the transcription factor-EB (TFEB), nuclear translocation and lysosome acidification, which could result in reduction of autophagic clearance, accumulation of lysosomal proteases and impairment of lysosome biogenesis [25]. This might be involved in inefficient mitochondrial turnover, impairing mitochondrial function that could lead to petite formation in yeast [26]. In neuronal cells, this could result in cell death or a cell division signal, both of which are lethal [27,28]. It could also be possible that this condition in neuronal cells could lead to aggregation of misfolded proteins, creating an even worse neuronal intracellular environment [29]. Impaired autophagy might also be responsible for the decreased protein clearance and lipid imbalances in the neuronal cells of AD patients, resulting in lipid dyshomeostasis and disruption in the proteostasis network [30]. Additionally, iron-binding sites in the N-terminus of Aβ42, increased intracellular iron levels in AD patients and the presence of iron magnetite nanoparticles in senile amyloid plaques of AD patients implicate possible complications due to iron accumulation [22]. Iron overloading and its reaction with hydrogen peroxide produced by MAO activity could further increase ROS levels [31]. Patients with AD may accumulate more intracellular Aβ in such conditions as iron overloading upregulates APP expression and processing [32], which could result in higher mitochondrial damage. Higher levels of the oxidative mitochondrial damage and impairment of mitophagy could also lead to lipofuscin formation, which could exacerbate cell survival [33,34].

The findings suggest the synergistic toxic effect of Aβ42 and tyramine, which was found to impair mitochondrial functioning due to hyper-induced oxidative damage in yeast cells. In relation to AD, these findings suggest important links between the trace amine and Aβ42, implying a possible crucial role of tyramine in brains of patients. Further studies to unveil the role of tyramine and other trace amines produced from tyramine including methyltyramine and *p*-octopamine could be important. Furthermore, tyramine present in the diet is of limited importance as tyramine does not cross the blood–brain barrier. However, possible transport of the precursor of tyramine to the brain should be evaluated. Studies suggest lower levels of tyrosine in the brains of AD patients, which could be due to its conversion to tyramine and other biogenic amines. Studies involving evaluation of tyramine levels in brains of AD patients could provide substantial evidence of its role in AD pathology. Possibilities of synergistic toxicity between low levels of tyramine and high levels of Aβ42 should not be ignored.

## 5. Conclusions

In summary, tyramine increased intracellular ROS in a dose-dependent manner, which was enhanced significantly in the presence of Aβ42, showing synergistic effect in yeast cells. Additionally, this collaborated toxicity was found to be detrimental in yeast cells, suggesting a possible novel cause of enhanced impairment of mitochondrial function and turnover in AD pathology.

## References

[1] Gainetdinov R.R., Hoener M.C., Berry M.D. (2018). Trace amines and their receptors. Pharmacol. Rev..

[2] Rutigliano G., Bräunig J., Grande C.D., Carnicelli V., Masci I., Merlino S., Kleinau G., Tessieri L., Pardossi S., Paisdzior S. (2019). Non-functional trace amine-associated receptor 1 variants in patients with mental disorders. Front. Pharmacol..

[3] Harmeier A., Obermueller S., Meyer C.A., Revel F.G., Buchy D., Chaboz S., Dernick G., Wettstein J.G., Iglesias A., Rolink A. (2015). Trace amine-associated receptor 1 activation silences GSK3β signaling of TAAR1 and D2R heteromers. Eur. Neuropsychopharmacol..

[4] Hauptmann N., Grimsby J., Shih J.C., Cadenas E. (1996). The metabolism of tyramine by monoamine oxidase A/B causes oxidative damage to mitochondrial DNA. Arch. Biochem. Biophys..

[5] Sherwani S.I., Khan H.A. (2016). Trace Amines in Neuropsychiatric Disorders. Trace Amines and Neurological Disorders: Potential Mechanisms and Risk Factors.

[6] Revel F.G., Moreau J.L., Gainetdinov R.R., Bradaia A., Sotnikova T.D., Mory R., Durkin S., Zbinden K.G., Norcross R., Meyer C.A. (2011). TAAR1 activation modulates monoaminergic neurotransmission, preventing hyperdopaminergic and hypoglutamatergic activity. Proc. Natl. Acad. Sci. USA.

[7] Cohen G., Farooqui R., Kesler N. (1997). Parkinson disease: A new link between monoamine oxidase and mitochondrial electron flow. Proc. Natl. Acad. Sci. USA.

[8] Cadenas E., Davies K.J.A. (2000). Mitochondrial free radical generation, oxidative stress, and aging. Free Radic. Biol. Med..

[9] Valoti M., Morón J.A., Benocci A., Sgaragli G., Unzeta M. (1998). Evidence of a coupled mechanism between monoamine oxidase and peroxidase in the metabolism of tyramine by rat intestinal mitochondria. Biochem. Pharmacol..

[10] Akanuma S.I., Yamazaki Y., Kubo Y., Hosoya K.I. (2018). Role of cationic drug-sensitive transport systems at the blood-cerebrospinal fluid barrier in para-tyramine elimination from rat brain. Fluids Barriers CNS.

[11] Roberts B.R., Lind M., Wagen A.Z., Rembach A., Frugier T., Li Q.-X., Ryan T.M., McLean C.A., Doecke J.D., Rowe C.C. (2017). Biochemically-defined pools of amyloid-β in sporadic Alzheimer’s disease: Correlation with amyloid PET. Brain.

[12] Chen X., Petranovic D. (2015). Amyloid-β peptide-induced cytotoxicity and mitochondrial dysfunction in yeast. FEMS Yeast Res..

[13] Murphy M.P., LeVine H. (2010). Alzheimer’s disease and the amyloid-beta peptide. J. Alzheimers Dis..

[14] Wolozin B., Wang S.W., Li N.-C., Lee A., Lee T.A., Kazis L.E. (2007). Simvastatin is associated with a reduced incidence of dementia and Parkinson’s disease. BMC Med..

[15] Dhakal S., Subhan M., Fraser J.M., Gardiner K., Macreadie I. (2019). Simvastatin Efficiently Reduces Levels of Alzheimer’s Amyloid Beta in Yeast. Int. J. Mol. Sci..

[16] Dhakal S., Kushairi N., Phan C.W., Adhikari B., Sabaratnam V., Macreadie I. (2019). Dietary Polyphenols: A Multifactorial Strategy to Target Alzheimer’s Disease. Int. J. Mol. Sci..

[17] Caine J., Sankovich S., Antony H., Waddington L., Macreadie P., Varghese J., Macreadie I. (2007). Alzheimer’s Aβ fused to green fluorescent protein induces growth stress and a heat shock response. FEMS Yeast Res..

[18] Porzoor A., Alford B., Hügel H.M., Grando D., Caine J., Macreadie I. (2015). Anti-amyloidogenic properties of some phenolic compounds. Biomolecules.

[19] Almshawit H., Macreadie I. (2017). Fungicidal effect of thymoquinone involves generation of oxidative stress in *Candida glabrata*. Microbiol. Res..

[20] Khan M.Z., Nawaz W. (2016). The emerging roles of human trace amines and human trace amine-associated receptors (hTAARs) in central nervous system. Biomed. Pharmacother..

[21] Farooqui T., Farooqui A.A. (2016). Trace Amines and Neurological Disorders: Potential Mechanisms and Risk Factors.

[22] Liu J.L., Fan Y.G., Yang Z.S., Wang Z.Y., Guo C. (2018). Iron and Alzheimer’s Disease: From Pathogenesis to Therapeutic Implications. Front. Neurosci..

[23] Prakash A., Dhaliwal G.K., Kumar P., Majeed A.B.A. (2017). Brain biometals and Alzheimer’s disease–boon or bane?. Int. J. Neurosci..

[24] Kim A.C., Lim S., Kim Y.K. (2018). Metal Ion Effects on Aβ and Tau Aggregation. Int. J. Mol. Sci..

[25] Santin Y., Sicard P., Vigneron F., Guilbeau-Frugier C., Dutaur M., Lairez O., Couderc B., Manni D., Korolchuk V.I., Lezoualc’H F. (2016). Oxidative Stress by Monoamine Oxidase-A Impairs Transcription Factor EB Activation and Autophagosome Clearance, Leading to Cardiomyocyte Necrosis and Heart Failure. Antioxid. Redox Signal..

[26] Kerr J.S., Adriaanse B.A., Greig N.H., Mattson M.P., Cader M.Z., Bohr V.A., Fang E.F. (2017). Mitophagy and Alzheimer’s Disease: Cellular and Molecular Mechanisms. Trends Neurosci..

[27] Akbar M., Essa M.M., Daradkeh G., Abdelmegeed M.A., Choi Y., Mahmood L., Song B.J. (2016). Mitochondrial dysfunction and cell death in neurodegenerative diseases through nitroxidative stress. Brain Res..

[28] Kubli D.A., Gustafsson Å.B. (2012). Mitochondria and mitophagy: The yin and yang of cell death control. Circ. Res..

[29] Chakravorty A., Jetto C.T., Manjithaya R. (2019). Dysfunctional mitochondria and mitophagy as drivers of Alzheimer’s disease pathogenesis. Front. Aging Neurosci..

[30] Zhang X., Evans T.D., Jeong S.-J., Razani B. (2018). Classical and alternative roles for autophagy in lipid metabolism. Curr. Opin. Lipidol..

[31] Youdim M.B.H. (2018). Monoamine oxidase inhibitors, and iron chelators in depressive illness and neurodegenerative diseases. J. Neural Transm..

[32] Ndayisaba A., Kaindlstorfer C., Wenning G.K. (2019). Iron in Neurodegeneration–Cause or Consequence?. Front. Neurosci..

[33] Terman A., Dalen H., Eaton J.W., Neuzil J., Brunk U.T. (2004). Aging of cardiac myocytes in culture: Oxidative stress, lipofuscin accumulation, and mitochondrial turnover. Ann. N. Y. Acad. Sci..

[34] Keller J.N., Dimayuga E., Chen Q., Thorpe J., Gee J., Ding Q. (2004). Autophagy, proteasomes, lipofuscin, and oxidative stress in the aging brain. Int. J. Biochem. Cell Biol..

